# Evidence and implications of direct charge excitation as the dominant mechanism in plasmon-mediated photocatalysis

**DOI:** 10.1038/ncomms10545

**Published:** 2016-01-28

**Authors:** Calvin Boerigter, Robert Campana, Matthew Morabito, Suljo Linic

**Affiliations:** 1 Department of Chemical Engineering, University of Michigan, Arbor, Michigan 48109, USA

## Abstract

Plasmonic metal nanoparticles enhance chemical reactions on their surface when illuminated with light of particular frequencies. It has been shown that these processes are driven by excitation of localized surface plasmon resonance (LSPR). The interaction of LSPR with adsorbate orbitals can lead to the injection of energized charge carriers into the adsorbate, which can result in chemical transformations. The mechanism of the charge injection process (and role of LSPR) is not well understood. Here we shed light on the specifics of this mechanism by coupling optical characterization methods, mainly wavelength-dependent Stokes and anti-Stokes SERS, with kinetic analysis of photocatalytic reactions in an Ag nanocube–methylene blue plasmonic system. We propose that localized LSPR-induced electric fields result in a direct charge transfer within the molecule–adsorbate system. These observations provide a foundation for the development of plasmonic catalysts that can selectively activate targeted chemical bonds, since the mechanism allows for tuning plasmonic nanomaterials in such a way that illumination can selectively enhance desired chemical pathways.

## Abstract (web summary)

The excitation of metal nanoparticles with light can lead to localized surface plasmon resonances, capable of driving chemical reactions in bound species. Here, the authors elucidate this mechanism and suggest that future plasmonic catalysts may be able to selectively activate specific chemical bonds.

## Graphical abstract

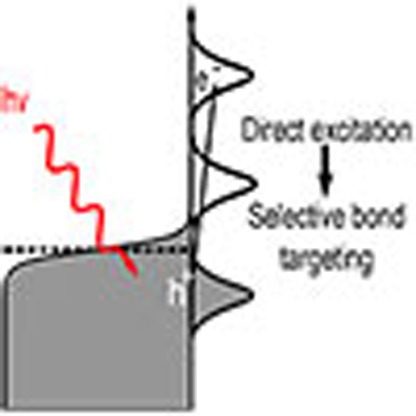

Numerous studies have shown that plasmonic metal nanoparticles can drive photochemical reactions directly on the surface of the nanoparticles when illuminated with relatively low-intensity light of particular frequencies^1^,^2^,^3^,^4^. It has been proposed that these processes are driven by the excitation of localized surface plasmon resonance (LSPR) on the metal nanoparticles^4^,^5^. LSPR is the resonant collective oscillation of valence electrons, established when the frequency of an incident electromagnetic field matches the natural frequency of surface electrons oscillating against the restoring force of positive nuclei^6^. It has been proposed that the interaction of LSPR with energetically accessible adsorbate orbitals can yield a charge injection process, where energized charge carriers (electrons or holes, formed by the plasmon decay) transiently populate otherwise unpopulated electronic states (orbitals), centred on the adsorbate molecule^7^,^8^,^9^. In this process, the adsorbate (more specifically, the entire adsorbate–nanoparticle system) is moved to a different potential energy surface and forces are induced on atoms in the adsorbate. These forces lead to nuclear motion of atoms in the adsorbate, which can result in the activation of chemical bonds and chemical transformations^10^. This mechanism of charge-driven chemical transformations is known by its original name ‘desorption (reaction) induced by electronic transitions'.

The mechanism of the charge injection process on excited plasmonic metal nanoparticles and the role of LSPR in the process are not well understood. The illustration in Fig. 1 outlines the two potential microscopic mechanisms by which charge injection can occur: indirect and direct. The indirect mechanism assumes that charge carriers are excited from occupied states to higher-energy unoccupied states within the metal nanoparticle yielding an excited electron distribution. Subsequently, charge carriers of appropriate energy from this distribution can scatter through the adsorbate states (orbitals) forming transiently charged adsorbates. The role of LSPR in this process is to increase the rates of charge excitation, and the energetic electron distribution associated with this mechanism is very similar to the one obtained if a high-intensity laser was illuminated on an extended metal structure. On the other hand, the direct transfer mechanism assumes the direct LSPR-induced electron excitation from occupied to unoccupied orbitals of the molecule–nanoparticle complex (that is, the excitation process) is not mediated by the formation of an excited electron distribution within the metal nanoparticle. Instead, the decay of an oscillating surface plasmon results in the excitation of an electron directly into an unoccupied adsorbate orbital of matching energy. The direct and indirect charge excitation processes, as well as nanoparticle heating due to photon absorption can all theoretically result in photochemistry, and it is not clear which of these processes is dominant^11^.

The identification of the dominant mechanism of charge excitation and the impact of LSPR is highly consequential in answering the question whether plasmonic nanostructures can be tuned in a way that under illumination they selectively enhance particular chemical pathways, while suppressing other chemical pathways. As illustrated in Fig. 1b, if indirect charge transfer plays the dominant role then the steady-state concentration of energetic electrons is the highest close to the Fermi level (that is, similar to Fermi–Dirac electron distribution) and therefore the electron-driven chemical transformation would preferentially proceed through the adsorbate orbitals closest to the Fermi level. This mechanism does not offer much opportunity to tune the chemical pathways by tuning the plasmonic properties of nanostructures since LSPR would only enhance the rates of electron excitation without impacting the electron distribution. On the other hand, the direct mechanism assumes that the LSPR-mediated photon absorption occurs through the creation of electron–hole pairs of specific energy that are localized on the adsorbate as illustrated in Fig. 1a. The direct mechanism offers an opportunity to affect chemical selectivity by creating plasmonic nanostructures with the LSPR characteristics that enhance the rates of targeted electronic transitions and therefore the rates of chemical transformations that proceed through only those specific transitions.

Herein, we shed light on the underlying physical mechanisms involved in the LSPR-induced charge transfer and chemical transformations on optically excited plasmonic metal nanoparticles. To accomplish this, we have coupled optical characterization methods, mainly wavelength-dependent Stokes and anti-Stokes surface-enhanced Raman spectroscopy (SERS) of probe molecules chemisorbed on plasmonic Ag nanostructures, with the kinetic analysis of the rates of photocatalytic reactions of these molecules in otherwise inert environment. We demonstrate that on optically excited plasmonic nanoparticles, the direct charge transfer mechanism plays an important role and that it results in accelerated rates of chemical decomposition (or desorption) of a probe molecule in an inert atmosphere. We propose that strong localized LSPR-induced electric fields result in photon absorption through the direct excitation of an electron from specific occupied to unoccupied orbitals of the molecule–nanoparticle complex (that is, at the surface of the nanoparticle). This can induce photochemical transformation of the chemisorbed molecules via a desorption (or reaction) induced by electronic transitions mechanism described above^7^. We also discuss the potential impact of these findings and suggest ways to develop plasmonic catalysts that can potentially selectively activate targeted chemical bonds.

It is important to put our results in the context of numerous previous SERS studies of probe molecules on plasmonic nanoparticles. These previous studies have given us a wealth of information about various mechanisms and consequences of surface enhancement in Raman spectroscopy, even demonstrating that laser-induced electron-driven reactions on plasmonic metals are possible^9^,^12^,^13^. The results presented herein build upon these findings by focusing on the missing link between LSPR and charge excitation. We distinguish between the above-discussed two LSPR-mediated mechanisms of charge excitation by analysing the unique anti-Stokes spectral behaviour that is interpreted in terms of LSPR-mediated vibroelectronic excitation.

## Results

### Optical properties of the Ag nanocube SERS platform

The SERS platforms (that is, the model system) used in our studies contained small aggregate clusters of silver (Ag) nanocubes with the cube length of ∼75 nm dispersed on a silicon substrate as shown in the optical micrograph in the inset of Fig. 2. We perform SERS measurements of probe molecules using two different lasers with the wavelengths of 532 and 785 nm. The optical excitation of LSPR at these wavelengths results in high-intensity electric fields at the surface of the Ag nanoparticles. Since these local fields facilitate the photophysical and photochemical processes studied herein, we first employed classical finite difference time domain (FDTD) electrodynamics simulations to compute the local near field electric field intensities of Ag systems that modelled our SERS platform. These simulations serve to provide part of the framework with which to interpret the key Raman results in this study.

In our FDTD studies we, used model systems consisting of two and three Ag nanocubes positioned at different configurations at 1 and 2 nm separations from each other. Not surprisingly, the FDTD simulations show that the highest plasmonic electric field intensities are generated in the gaps between the nanoparticles (Fig. 2). We find that for both laser source wavelengths, the field intensity enhancements in the 1-nm gap between the two nanoparticles are similar to each other ((|**E|/|E**_**0**_**|**)^2^∼4 × 10^4^). Furthermore, we have also integrated the field intensity enhancement across the entire surface of the two nanoparticle system to obtain the average surface field intensity enhancements (Supplementary Table 1). We find that the average field intensity enhancements are within an order of magnitude of each other for the 532- and 785-nm sources. Similar analysis for the Ag nanoparticles separated by 2 nm from each other, as well as for the systems containing aggregates of three Ag nanoparticles, showed very similar results (Supplementary Figures 1–6, Supplementary Table 2, Supplementary Note 1).

It should be noted that the geometries simulated in the FDTD studies are idealized representation of our SERS platforms, which in practice contain aggregates of multiple nanoparticles with varying orientations and separation distances. The effect of particle orientations and separation distances on the optical properties of collections of plasmonic nanoparticles has been studied extensively previously. These studies showed that the resonant wavelength of the coupled dimer plasmon resonance peak is dependent on the particle separation, moving to longer wavelengths and higher maximum field intensities for smaller separations^14^,^15^. Therefore, due to the random nature of the Ag nanoparticle clustering in our system, we expect that there are different spots that support plasmon resonance peaks over a broad range of wavelengths. The data in Fig. 2a, which show the ultraviolet–visible extinction spectrum of a sample of silver nanocube aggregates used in the SERS studies, show this precise signature with a very broad extinction peak ranging between 600 and 900 nm. The slightly stronger and more confined extinction peak ranging from 450 to 550 nm is the result of dipolar LSPR within single particles, while still higher-energy peaks come from higher-order multipole resonances within individual particles^16^,^17^. The wavelengths of the Raman lasers used in our studies are overlaid on the spectrum.

It is worth noting that in addition to the above described photophysical properties, which can be captured by classical electrodynamics, there have been reports of quantum charge tunnelling between optically excited plasmonic nanoparticles^18^,^19^. This charge tunnelling process has been reported for the particle separations smaller than ∼0.3–0.5 nm (refs 20, 21, 22). It is a consequence of very large electric fields confined in the nanogaps that force the movement of charge between the particles. At these extremely small gap distances, the tunnelling process broadens and slightly blue shifts the LSPR resonance peaks while decreasing the maximum field intensity from the classical prediction.

To summarize, our analysis of the photophysical properties of the SERS platforms suggests that (i) there are sites in our system that can support LSPR with significantly elevated electric field intensities at both Raman laser wavelengths, (ii) while we know the field intensities are significantly elevated, we cannot know the exact intensity at each site due to the disordered nature of Ag aggregates in our SERS platforms and (iii) potential quantum tunnelling could be taking place in aggregates with interparticle gaps of 0.5 nm or less. With regards to the final two points, our analysis of Raman results and the conclusions drawn are not affected by the lack of exact knowledge of field intensities at every site in the system. By focusing our analysis on the ratios of anti-Stokes to Stokes signals in a given measurement and probing the dependence of only these ratios on the incident photon intensity, we find the exact value of the electric field intensity to be irrelevant so long as it is strong enough to support SERS.

### SERS measurements for methylene blue on Ag

There have been a few studies of the chemisorption of methylene blue (MB) and similar molecules on Ag surfaces^23^,^24^,^25^. It has been suggested that MB chemisorbs through a covalent chemical bond between an Ag surface atom and the nitrogen atom in the aromatic ring of MB. We have also performed density functional theory (DFT) quantum calculations of adsorbed thiazine on Ag model slabs. We use thiazine as a surrogate for MB since similar to MB, thiazine has an N- and S-containing aromatic ring and similar bonding conformations are proposed. Thiazine is smaller than MB and therefore more easily handled computationally. Our DFT calculations, using the revised Perdew-Burke-Ernzerhof (RPBE) density functional, showed that the binding energy of thiazine to the Ag(100) surface facet is −0.29 eV (exothermic), while on the under-coordinated Ag(211) surface the binding energy is −0.37 eV. Since the RPBE functional in general underestimates the binding electronic energy and neglects van der Waals forces, we suggest that in our systems MB chemisorbs on the surface sites of Ag nanocubes, essentially coating the nanoparticles.

There are two established potential mechanisms by which LSPR can enhance Raman scattering. One mechanism involves the LSPR-induced enhancement of the local electric field intensity at the adsorbate (electromagnetic mechanism) leading to the enhancement of the Raman signal. The electromagnetic enhancement factor (Σ) of Raman signal depends on electromagnetic field enhancement at the location occupied by a probe molecule. It can be closely approximated by:


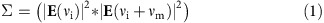


where **E**(*ν*) is the local electromagnetic field enhancement at a photon frequency *ν*. *ν*_i_ and (*ν*_i_+*ν*_m_) are the frequencies of the incident (that is, laser) photon and scattered (that is, Raman shifted) photon, respectively^26^,^27^. The other mechanism involves the LSPR-induced charge excitation (or charge–exchange) mechanism^27^. Charge excitation SERS occurs as a result of the LSPR-mediated charge excitation from filled to unfilled electronic states of the nanoparticle–adsorbate complex (one or both of these electronic states are localized on the adsorbate). The propensity to undergo charge excitation is, among other things, determined by the local electronic structure of the adsorbate–metal complex^28^.

Since we were interested in investigating the LSPR-induced charge excitation process and the chemical consequences of this process, we focused on identifying systems where these two mechanisms can be separated from each other, centring our studies on the charge excitation mechanism and probing its effect on chemical transformations. Below, we demonstrate that MB on Ag exhibits a LSPR-mediated charge excitation only at specific excitation wavelengths and that this process increases the rates of chemical reactions (in this case the degradation or desorption of MB on the Ag surface). These observations allow us to conclude that in this case LSPR mediates the process of charge excitation into adsorbates via the above-discussed direct mechanism.

The process of charge excitation within the metal–adsorbate complex has some key experimentally distinguishable SERS signatures. These signatures are a consequence of the fact that the charge excitation process results in an elevated population of excited vibrational modes compared with the equilibrated, thermalized system. Therefore, a key signature of the charge transfer process is an elevated anti-Stokes Raman intensity compared with that expected for a thermodynamically equilibrated system. We note that Stokes scattering signal in Raman spectroscopy is the measure of the rate of transitions of a molecule from the ground to the first excited vibrational states, while the anti-Stokes scattering signal measures the reverse process. The ratio of anti-Stokes to Stokes scattering rates and thus their signal intensities for a vibrational mode can be related to the population of vibrationally excited molecules^29^.

Data in Fig. 3 show Stokes and anti-Stokes SERS spectra for the Ag–MB complex at 532 nm (Fig. 3a) and 785 nm (Fig. 3b) excitation wavelengths. The spectra were collected using the same photon flux of 2.0 × 10^20^ cm^−2^ s^−1^ for both lasers. Visual inspection of the data in Fig. 3 shows that the ratio of anti-Stokes to Stokes signals at 785 nm is higher than when the 532 nm laser source was used. To quantify these observations, the ratio of anti-Stokes/Stokes signal (*ρ*_SERS_) for specific MB vibrational modes (*ρ*_m_) of the Ag–MB complex under 785 and 532 nm excitations was computed and compared with the ratio for a similar energy mode in liquid toluene (*ρ*_tol_). Using toluene as an external standard corrects for any wavelength-dependent biases due to the sensitivity of the charge-coupled device detector, as demonstrated previously^30^,^31^. Liquid toluene gives a basic non-SERS, non-resonant Raman spectrum at visible light excitation, and the anti-Stokes to Stokes intensity ratios in toluene are consistent with those predicted by Boltzmann thermal distribution^30^. The degree by which the SERS anti-Stokes signal exceeded the expectation of the Boltzmann distribution is described through the quantity (*K*), which was calculated using the following equation:


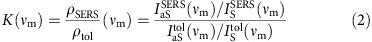


where 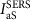
and 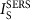
are the measured anti-Stokes and Stokes intensities, respectively, for MB on Ag at a given vibrational mode energy (*ν*_m_). 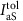 and 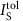 are the anti-Stokes and Stokes intensities, for the similar vibrational mode in toluene.

The data in Table 1 show the calculated *K* values for various modes of the Ag–MB complex at 532- and 785-nm excitation at the photon flux of 2.0 × 10^20^ cm^−2^ s^−1^. The data show that the *K* values under 785-nm excitation range between 4 and 34 for different Ag–MB modes. On the other hand, the *K* values for the 532-nm excitation are close to 1. The elevated *K* values at 785 nm compared with 532 nm suggest that charge excitation might be taking place within the Ag–MB complex at 785 nm and that there is no charge excitation for 532-nm wavelength.

We must note however that the SERS enhancement of Stokes and anti-Stokes scattering, in addition to the vibrational populations, also depends on the field intensity at the frequency of scattered (that is, Raman shifted) photons as described in equation (1). Different field intensities at the frequencies of Stokes and anti-Stokes scattered photons can contribute to the elevated *K* values even without charge transfer^32^,^33^. To rule out this possibility, we have performed SERS studies using the same Ag nanocube SERS platform with other probe molecules (Rhodamine 6G and Acridine Orange), which under 785-nm radiation do not exhibit charge excitation (Supplementary Figure 7, Supplementary Table 3, Supplementary Note 2)^31^. These studies show that for these molecules the *K* values at 785 nm are significantly lower than the *K* values for MB, signalling that the elevated vibrational population observed for the Ag–MB complex at 785 nm is due to the charge excitation process.

Further confirmation of charge excitation taking place at 785 nm comes from observing that the ratio of anti-Stokes to Stokes signal shows a positive dependence on incident photon intensity. This is another key feature of the charge excitation process^29^,^34^. As discussed above, charge excitation can lead to a non-thermalized population of vibrationally excited molecules (that is, an elevated population of molecules in the first excited vibrational states, which are the initial states for *ρ*=1→0 transitions associated with anti-Stokes scattering). As the source intensity increases, probe molecules will have a larger fraction of modes that are excited to the first excited vibrational state and above, thus increasing the anti-Stokes to Stokes signal ratio^29^,^30^. This phenomenon is known as optical pumping, and in this regime the theoretically derived ratio (*ρ*) of anti-Stokes to Stokes signals is governed by the equation^29^:


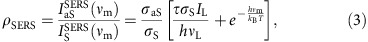


where *σ*_as_ and *σ*_s_ represent the cross-section for anti-Stokes and Stokes events respectively, *τ* the vibrational relaxation time constant for the molecule, *I*_L_ the incident light intensity, *hv*_L_ and *hv*_m_ the energy of the incident photon and vibrational mode respectively, at *T* the sample temperature.

The equation covers both the exponential dependence of the population of the excited vibrational states on temperature and the linear dependence on photon intensity (*I*_L_). In the optical pumping regime (that is, when the linear term in equation (3) is of the same order or greater than the exponential term) under constant sample temperature, *ρ*_SERS_ and thus *K* (as described above, the measure of the deviations in the anti-Stokes to Stokes ratios from the ratio predicted by the equilibrium Boltzmann distribution) will show a linear dependence on the photon intensity. The data in Fig. 4 show the *K* values plotted against incident photon intensity for some of the most prominent bands in the Ag–MB Raman spectrum under 785-nm excitation. The intensity spans two orders of magnitude of photon flux, from 1.5 × 10^19^ to 2.0 × 10^21^ cm^−2^ s^−1^. The data show a linearly increasing *K* for all modes as photon flux increases, providing compelling evidence that the samples are well within the optical pumping charge excitation regime. This is in contrast to the other measured probe molecules which showed no linear increase in *K* values with increasing intensity (Supplementary Fig. 8).

## Discussion

The above-discussed data represent evidence that Ag–MB complexes experience charge excitation resonance under exposure to 785 nm incident photons, while the charge excitation process does not take place with 532-nm laser under identical photon fluxes. It is important to reiterate that these results cannot be explained only by the different values of the field intensities at these two wavelengths. If this was the case, any molecule could exhibit elevated anti-Stokes signals under 785-nm illumination. We have ruled out this possibility in the above-mentioned SERS studies of other probe molecules (Rhodamine 6G and Acridine Orange), which under 785-nm illumination do not exhibit charge excitation. Similarly, these studies of other probe molecules also rule out interparticle electron tunnelling experienced at very small particle separations as the only requirement for the above-mentioned charge transfer process, as if this were the case we would again expect no difference in the results when using other probe molecules.

**Figure 1:**
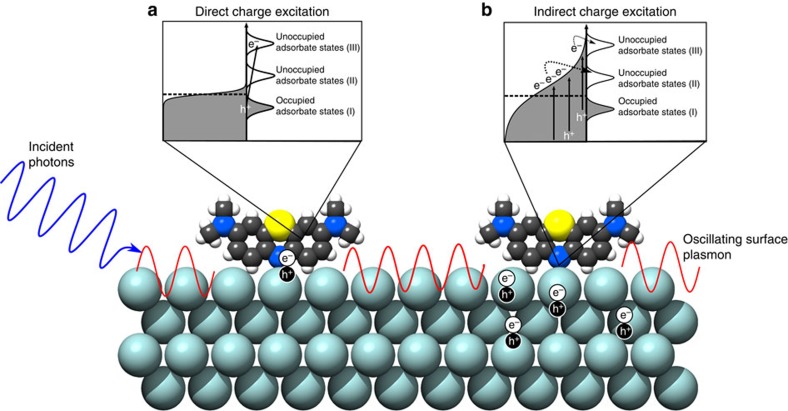
Illustration of LSPR-mediated charge excitation mechanisms. Incident photons excite oscillating surface plasmons on an adsorbate-covered Ag nanoparticle surface. These surface plasmon oscillations decay through the formation of energetic electron–hole pairs. In the direct process (**a**), the electron is excited directly into an unoccupied orbital of matching energy within the adsorbate. In the indirect process (**b**), the energetic electrons formed by decaying plasmons form a distribution within the metal nanoparticle. Electrons with proper energy can then scatter into available adsorbate orbitals. Because of the nature of the electron distribution formed in the indirect mechanism, more electrons will scatter into lower energy orbitals (II) and chemical transformation will preferentially proceed through that lower energy activated pathway. In the direct mechanism, however, the electrons can potentially excite into higher energy orbitals (III) when that energy matches the incident photon energy. This opens the possibility for selective chemical pathway targeting that impossible in the indirect mechanism.

**Figure 2:**
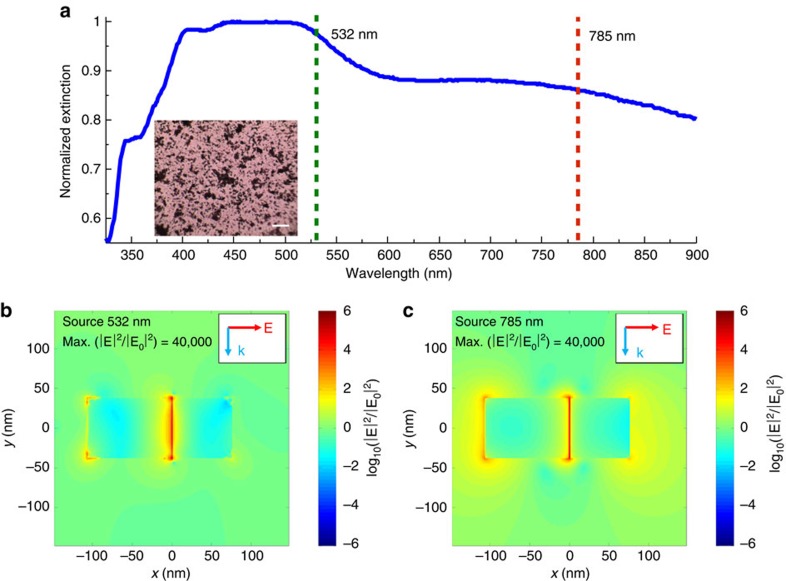
Optical characterization of Ag SERS platform. (**a**) Ultraviolet–visible spectrum of silver nanocubes after deposition onto solid substrate. The two dashed lines show the wavelengths of the lasers used in this study and their overlap with the LSPR of the silver cubes (inset: optical micrograph of Ag–MB sample showing high levels of aggregation; scale bar, 20 μm). (**b**,**c**) Simulated spatial distribution of electric field enhancement for a silver nanocube dimer (face-to-edge orientation) with separation distance of 1 nm under (**b**) 532 nm and (**c**) 785 nm photon excitation.

**Figure 3:**
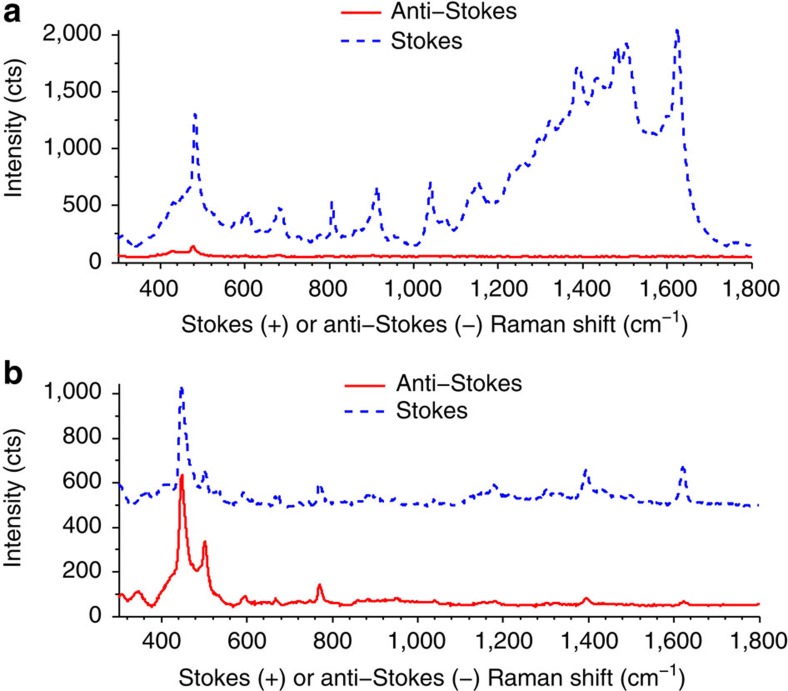
SERS spectra of silver nanocube-dye structures. (**a**,**b**) Stokes and anti-Stokes spectra for silver nanocube–methylene blue (Ag–MB) structures gathered using a 532-nm (**a**) or 785-nm (**b**) laser. All experimental parameters other than the wavelength of incoming photons, including incident photon flux, acquisition time and sample spot size were identical. It should be noted that Stokes shifts have positive wavenumbers (cm^−1^), while anti-Stokes shifts are negative. They are plotted on the same positive axis here to allow for direct comparison.

**Figure 4:**
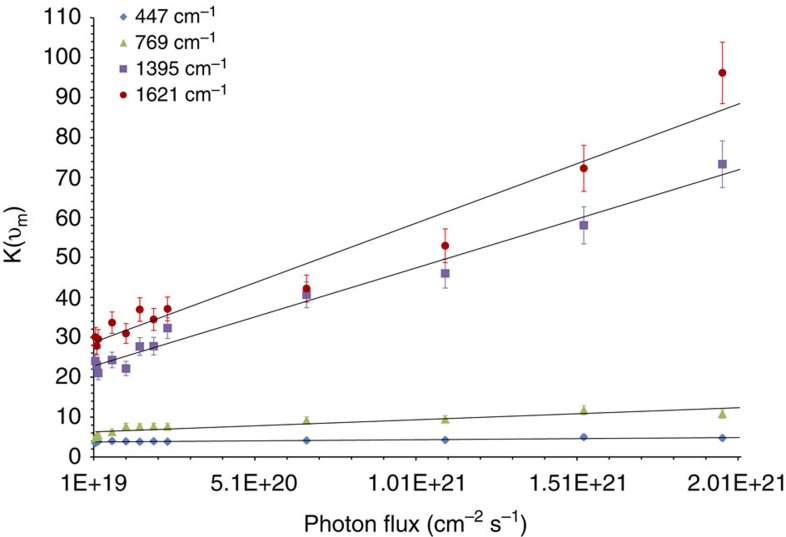
Excess anti-Stokes signal as a function of incident photon flux. Excess anti-Stokes signal ratio (*K*) dependence on the incident photon flux for multiple prominent bands in the methylene blue spectrum gathered using the 785-nm excitation wavelength. The linear nature of the dependence is compelling evidence that optical pumping of the MB molecules is occurring. Error bars represent s.d. of three separate experimentally gathered *K* values for a given band and photon flux.

The data presented above-shed light on some microscopic features of the charge excitation process, depicted in Fig. 1. We have postulated that the charge excitation can take place following an indirect route where excited charge carriers are first formed within the nanoparticle in a process that results in an excited electron distribution. Electrons from this distribution that gain sufficient energy can scatter through the adsorbate states forming transiently charged adsorbates. Conversely, in the direct excitation process, the presence of unoccupied adsorbate orbitals at a specific energy allows for a direct, resonant transition from occupied to unoccupied orbitals of the molecule–nanoparticle complex. We suggest that the occurrence of charge excitation only by photons of specific energy (785 nm) and not by those of higher energy (532 nm) under identical incident photon intensities shows that the mechanism of charge excitation in this system is direct. If the mechanism were indirect, one could expect to see evidence of charge excitation at any wavelength with photon energies at least as high as the difference between the metal Fermi level and the lowest unoccupied molecular orbitals of the adsorbate. Under the assumption of the indirect mechanism, as photon energies increased (for example, from 785 to 532 nm), the extent of charge excitation would be expected to rise, as the number of electrons within the metal that reached the requisite energy to scatter into the unfilled adsorbate states would increase. Because this does not occur and instead we observe charge excitation only under lower energy photon excitation, we suggest that the process of charge excitation is direct and it involves direct resonant single electron transitions from occupied to unoccupied states of the Ag–MB complex.

The evidence that points towards a direct mechanism of charge excitation also serves to rule out plasmon-induced heating of the nanoparticles as an explanation for the high-anti-Stokes data. The *K* values for the SERS data collected under 532-nm excitation are close to 1, suggesting that there is no significant laser induced heating of the entire system at this excitation wavelength. Equilibrated heating of the system would increase the population of excited vibrational modes of the molecule and result in elevated anti-Stokes intensity and therefore elevated *K* values. On the basis of the fact that for identical photon fluxes, the energy dissipated into the system would be higher for the 532 nm laser compared with the 785 laser, we argue that equilibrated heating of the entire sample does not take place for the 785-nm laser either. This suggests that the process of charge excitation observed at 785 nm results in energy being selectively deposited into the adsorbed molecule rather than into the entire system.

We can also speculate about the nature of the electronic states involved in the charge excitation process. The highest occupied molecular orbital–lowest unoccupied molecular orbital energy gap in the free MB molecule is ∼1.86 eV (665 nm), which is above the energy of the photons from the 785 nm (1.6 eV) laser. An examination of the absorption spectrum of aqueous MB shows no interaction with light near 785 nm. This means that the electronic states involved in the charge excitation processes are associated with either strongly perturbed states localized on MB—these perturbations can be due to the chemisorption of MB in the Ag metal—or with hybridized Ag–MB electronic states.

It is also important to discuss how LSPR could affect this process of charge excitation. As discussed above, our data point us in the direction of the direct one-electron excitation by 785 nm photons among the states within the Ag–MB complex. Clearly, the existence of the electronic states that can be accessed by the 785-nm photon is a requirement for the observed charge excitation process. The SERS data suggest that there are high LSPR-mediated electron transition rates between these states. The high transition rates can only be observed under the following conditions: first, there is a large density of the electronic states corresponding to these transitions. Second, there is a high degree of overlap between the corresponding wave functions of the associated states (giving large oscillator strengths). Third, there must exist a high-intensity electric field driving the excitation process (pushing electrons from the occupied to unoccupied states) at the location of the adsorbate. Whether the first and second requirements are fulfilled depends on the local electronic structure of the Ag–MB system, the properties of which are determined by the chemisorption of MB on Ag. We hypothesize that LSPR plays a role in the third requirement, that is, LSPR is providing high-intensity fields, particularly in the small interparticle gaps, at frequencies that can support the high rates of the electronic transitions between the states centred on the adsorbate.

This hypothesis that the role of LSPR is somewhat decoupled from the role of the local electronic structure has far reaching consequences in the potential design of selective plasmonic catalytic systems. It suggests that by engineering plasmonic nanomaterials with plasmon resonances that match the charge excitation energies of adsorbates interacting with the plasmonic nanostructure, it is possible to engineer systems that can support higher rates of electronic excitations between electronic states of a particular energy (at particular wavelengths). A natural implication of this hypothesis is that plasmonic nanomaterials can be tuned in such a way that under illumination they will selectively enhance particular chemical pathways (those which are activated by the particular electronic transitions supported by plasmon-induced local fields) while suppressing other chemical pathways. One way to achieve this objective might be to combine plasmonic particles with more active metals in a single catalyst^35^,^36^ or have bimetallic nanoparticles that combine a plasmonic metal core and a more chemically reactive metal shell. In these systems, the interaction of the chemically reactive metal with adsorbates allows for tuning of the electronic structure of adsorbates on the nanoparticle (based on the selection of the metal), while the optical properties are governed by the plasmonic metal. By matching these electronic and optical properties, it could be possible to induce preferential selective charge transfer to a particular orbital, essentially guiding photochemical transformation towards the more selective pathway.

**Figure 5:**
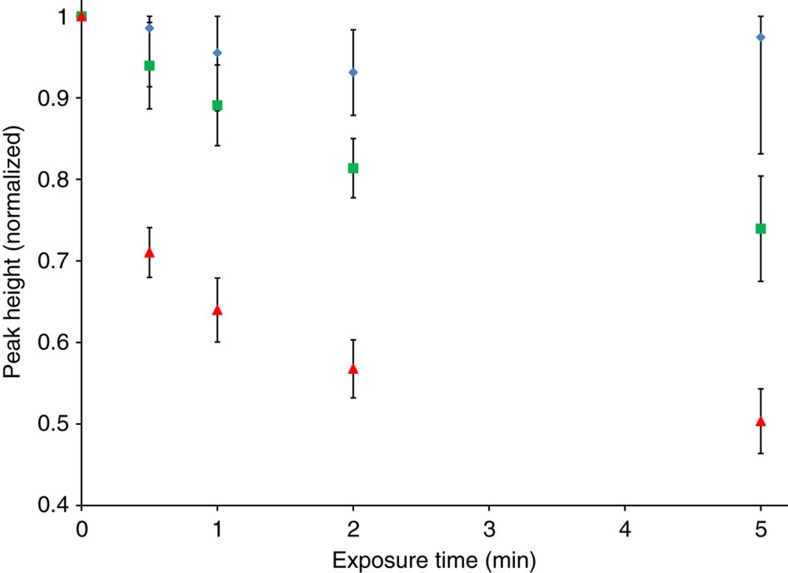
Methylene blue signal degradation by prolonged laser exposure. Normalized heights of the 447 cm^−1^ Raman mode of Ag–MB gathered at 373 K and in inert N_2_ atmosphere after exposure to no laser (control, blue diamonds), 532-nm laser (green squares) or 785-nm laser (red triangles) for a given periods of time. The 785-nm laser shows significant signal degradation enhancement compared with the 532 nm and control samples.

A direct corollary of the hypothesis that nanomaterials with plasmon resonance wavelengths matching the charge excitation energies of surface adsorbates can accelerate chemical reactions of the adsorbates at these wavelengths is that MB on Ag should be more reactive under 785-nm illumination than under 532 nm. To test the corollary, we measured the rates of decomposition/desorption of MB on the surface of the Ag nanoparticles illuminated with 2.0 × 10^20^ cm^−2^ s^−1^ photon flux of the 785- and 532-nm light sources, under inert N_2_ atmosphere and at constant 100 °C reactor temperature. Data in Fig. 5 show the differences in the extent of SERS signal degradation of the most prominent Raman mode in MB (447 cm^−1^) over a given period of laser exposure. The data show that under exposure to the 785-nm laser, MB signal degradation occurs significantly faster than under exposure to the 532-nm laser of identical intensity. The instantaneous rate (rate as time→0) of MB decomposition/desorption under 785-nm exposure was calculated to be 4.8 × higher than for MB under equal intensity 532-nm exposure.

It is worth noting that the signal degradation enhancement under the 532-nm laser was not zero. The high electromagnetic fields formed on the Ag surface under 532-nm irradiation can cause instabilities in surface adsorbates and localized heating of the samples above the controlled reactor temperature can potentially occur during the prolonged laser exposure required in these studies^37^. Adsorbate instability due to high electromagnetic fields and heating cannot however explain the higher 785 nm degradation rates compared with 532 nm. The higher 785 nm degradation rates suggest that the observed charge transfer, which we have shown leads to pumping of excited vibrational modes, yields more unstable and reactive adsorbate molecules.

In these studies, we have coupled optical characterization methods with kinetic analysis of the rates of photocatalytic reactions to study the interaction of a probe molecule with optically excited plasmonic Ag nanoparticles. Specifically, our measurements and analysis of wavelength-dependent anti-Stokes and Stokes Raman spectra and their connection to photocatalytic decomposition/desorption of MB on Ag provide unique insight into the specifics of the mechanism of plasmon-mediated photocatalysis. We demonstrated that in the systems where the LSPR-mediated charge transfer from nanoparticles to adsorbates takes place, this charge transfer process results in elevated rates of chemical transformation. Furthermore, we showed that the mechanism of charge transfer involves the action of strong localized LSPR-induced electric field that results in an energy-specific direct electron excitation from occupied to unoccupied orbitals of the molecule–nanoparticle complex. Our findings suggest that the role of LSPR is somewhat decoupled from the role of the local electronic structure in plasmonic photocatalysis, and that by engineering plasmonic nanomaterials with resonant wavelengths matching the charge excitation energies of adsorbates interacting with the plasmonic nanostructure, it is possible to design systems that can support very high rates of electronic excitations between electronic states of a particular energy. These observations provide a foundation for the development of plasmonic catalysts that can selectively activate targeted chemical bonds, since it is implied that plasmonic nanomaterials can be tuned in such a way that under illumination they selectively enhance particular chemical pathways.

## Methods

### Sample synthesis and experimental SERS studies

The plasmonic nanoparticles in these Raman studies were silver nanocubes, synthesized via the modified polyol method^38^. Synthesized cube samples were characterized by STEM imaging and found to have an average size of 71±9 nm. MB, Rhodamine 6G and Acridine Orange were used as probe molecules in model systems. After synthesis, the cubes were washed 5 × by centrifugation and redispersion in a combination of acetone and ethanol. After washing, the cubes were dispersed in ethanol and small amount of NaCl was added to aid aggregation of Ag nanoparticles during the drying process. One of the dye molecules was added to the liquid solution at a concentration of 40 μM and allowed to incubate for several hours to ensure full equilibrium adsorption of the dye onto the silver surface^39^. Adding the dyes while the particles are still in liquid solution allows the molecules to access all available adsorption sites on the Ag particles. Solid samples for spectroscopic studies were prepared by drop coating the solution onto a glass or silicon chip and allowing the ethanol solvent to evaporate, leaving behind a thin layer of silver-dye aggregates. Ultraviolet–visible spectra were gathered using samples on glass slides and a Thermo Evolution 300 spectrometer. Stokes and anti-Stokes Raman spectra were gathered using a Horiba LabRAM HR system under excitation from either a 532-nm diode-pumped solid state laser or a 785-nm diode laser. The acquisition time for the spectra was 2 s to negate heating effects due to prolonged laser exposure. In solid samples, the laser was focused on nanoparticle aggregates visible through a × 50 microscope lens. In liquid samples, including toluene used for standardizing expected signal ratios, the laser focus was near the surface of the liquid.

### Photocatalytic experimental studies

Photoreaction studies were conducted in a temperature-controlled Harrick Praying Mantis reaction chamber designed for Raman spectroscopy. A sample of MB on silver on a silicon chip was placed inside the chamber, with the transparent reactor window allowing Raman spectra to continue to be collected. The temperature of the sample within the reactor could then be controlled, and pure nitrogen could be flowed through the reactor to provide an inert atmosphere. Samples in MB degradation studies were heated to 373 K under N_2_ atmosphere and subsequently exposed to one of two lasers (532 or 785 nm, at equal photon flux) or no light (control) for a measured period of time. Losses in Raman peak signal for prominent bands in MB were measured and interpreted as decreases in surface coverage of MB due to chemical change (decomposition or desorption).

### FDTD simulations

FDTD simulations were carried out using the Meep simulation package^40^. The simulations were performed in a three-dimensional grid measuring 294 × 294 × 294 nm. An additional 50 nm of perfectly matched layer was added to all sides of this grid. The space between grid points was 1 nm for all but one simulation in which the much more computationally expensive 0.5-nm mesh was used to ensure that a shrinking mesh size did not change significantly the behaviour of the field intensities within gaps between particles. The dielectric function for silver was obtained from data published by Rakic *et al*^41^. Nanocube dimers were arranged in a number of orientations, including face to face, face to edge, edge to edge and corner to corner; each dimer system was tested with interparticle separations of both 1 and 2 nm. In addition, Ag nanocube trimers were also simulated in edge–face–edge and edge–edge–edge orientations with 1-nm separations. The source wavelengths studied matched those of the laser in the Raman set-up, 532 and 785 nm. The energy from the source propagated perpendicular to the interparticle axis; the polarization of the source was alternately perpendicular and parallel to the interparticle axis in separate simulations. Field enhancement data were visualized on the *x*–*y* and *x*–*z* planes. Integrated field values were calculated by finding the average value of the electric field data points located on the nanoparticle surfaces only.

### DFT simulations

DFT calculations were performed using real space grid-based projector augmented wavefunction DFT implemented in the GPAW software package^42^. The exchange–correlation interaction was approximated using the RPBE^43^,^44^. The Ag[100] and Ag[211] surfaces were modelled using 3 × 3 slabs and 2 × 3 slabs, respectively. All systems used four layer slabs, with 10 Å of vacuum space. Pyridine molecules were adsorbed vertically over a top site [100] or above a step site [211] to match the predominant facets present in the synthesized Ag nanocubes. Multiple adsorption configurations were tested for the molecules on the Ag surfaces.

## Additional information

**How to cite this article:** Boerigter, C. *et al*. Evidence and implications of direct charge excitation as the dominant mechanism in plasmon-mediated photocatalysis. *Nat. Commun.* 7:10545 doi: 10.1038/ncomms10545 (2016).

## Supplementary Material

Supplementary Information Supplementary Figures 1-8, Supplementary Tables 1-3, Supplementary Notes 1-2 and Supplementary References

## Figures and Tables

**Table 1:** *K* values in Ag–MB spectra.

**Peak (cm**^**−1**^**)**	***K***
532 nm	
482	0.63
807	0.6
914	1.24
1,041	1.32
	
785 nm	
447	3.91
771	7.67
1,394	27.75
1,621	34.45

MB, methylene blue; SERS, surface-enhanced Raman spectroscopy.

Calculated *K* values for selected peaks in the MB SERS spectrum excited by 532- and 785-nm lasers.
