# Supplementary material for: Evidence and implications of direct charge excitation as the dominant mechanism in plasmon-mediated photocatalysis
Source: Nat Commun. 2016 Jan 28;7:10545. doi: 10.1038/ncomms10545 (PMC4738363; doi:10.1038/ncomms10545)
Supplement: Supplementary Information — Supplementary Figures 1-8, Supplementary Tables 1-3, Supplementary Notes 1-2 and Supplementary References [file ncomms10545-s1.pdf]

Supplementary Figures

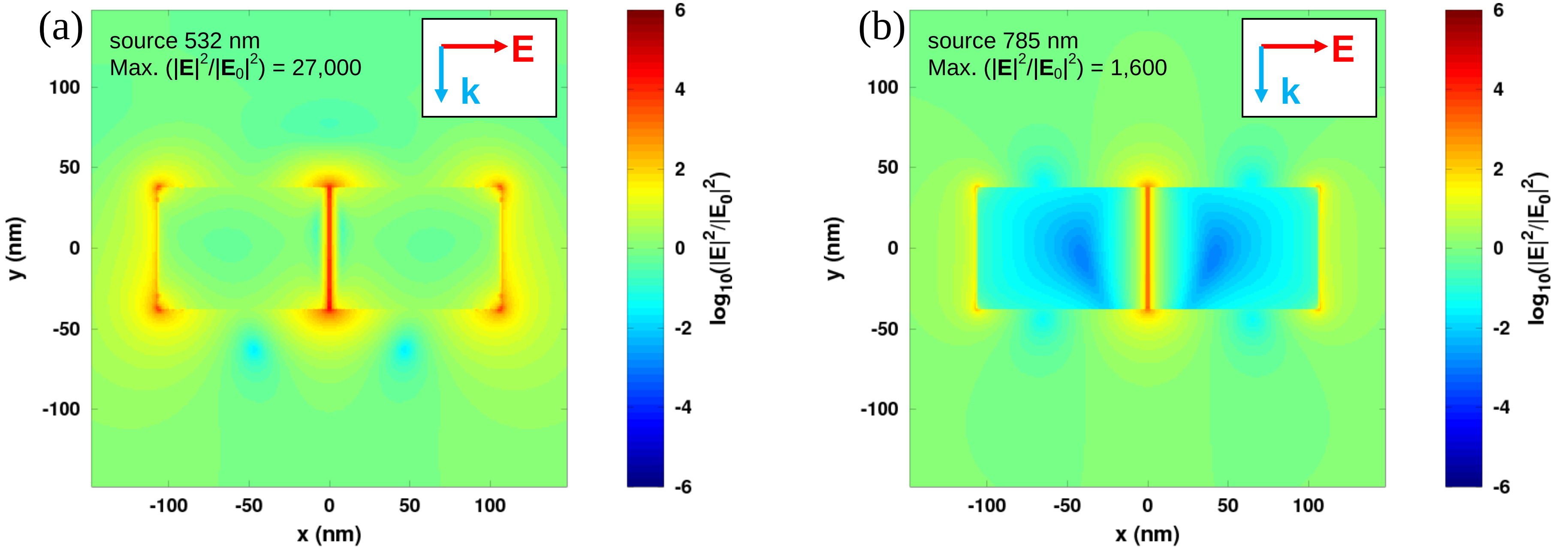

Supplementary Figure 1 – Dimer, edge-edge orientation, 1nm separation. Spatial electric field enhancement distribution (@532nm, (a), and @785nm, (b)) for a dimer of silver nanocubes (side length 75 nm), oriented edge to edge, inter-particle separation 1 nm, source polarized *parallel* to the inter-particle axis.

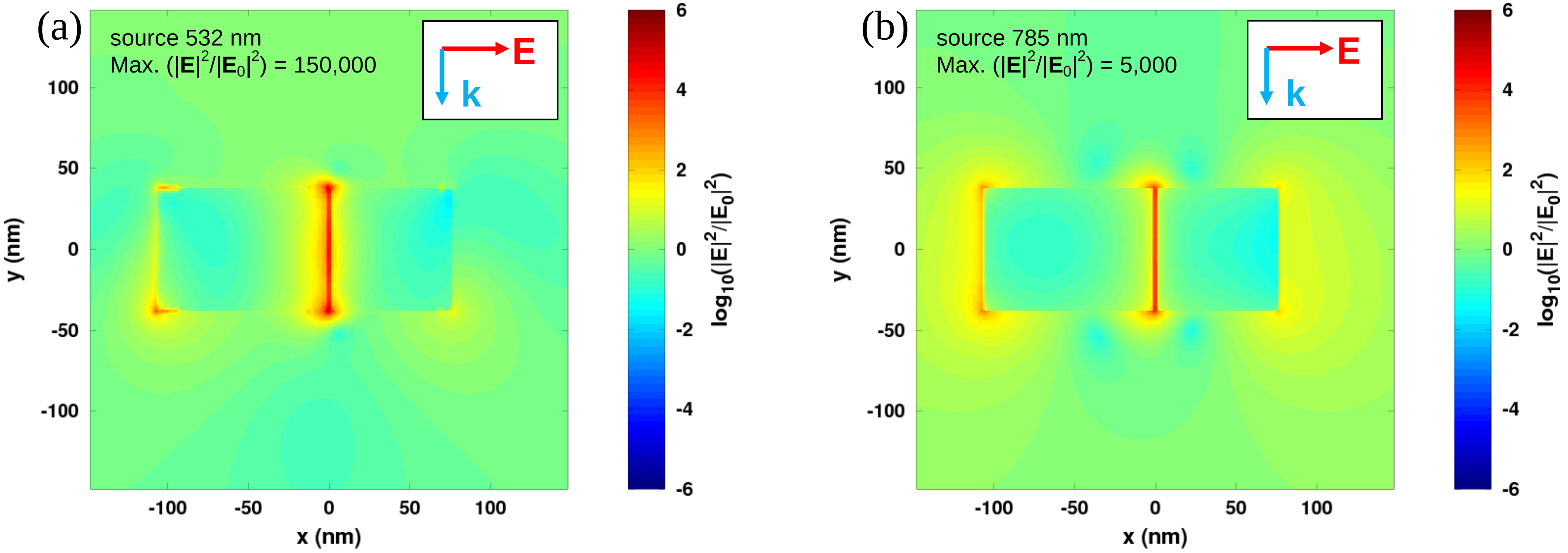

Supplementary Figure 2 - Dimer, face-edge orientation, 2nm separation. Spatial electric field enhancement distribution (@532nm, (a), and @785nm, (b)) for a dimer of silver nanocubes (side length 75 nm), oriented face to edge, inter-particle separation 2 nm, source polarized *parallel* to the inter-particle axis.

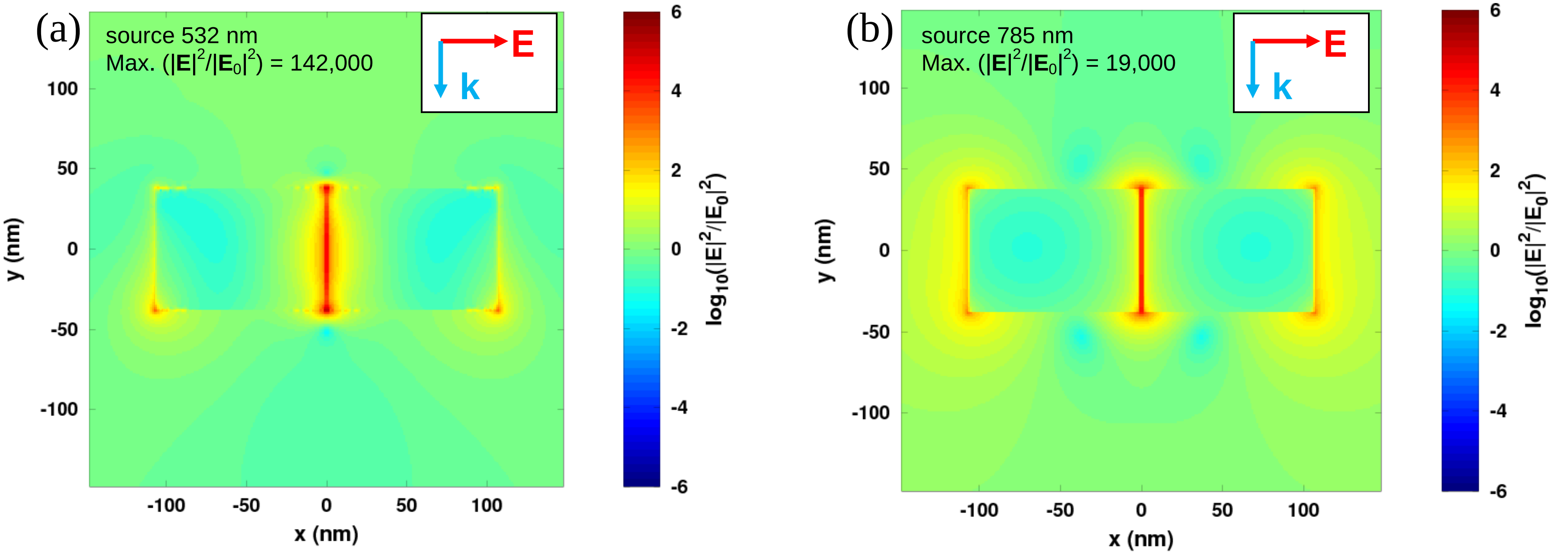

Supplementary Figure 3 - Dimer, edge-edge orientation, 2nm separation. Spatial electric field enhancement distribution (@532nm, (a), and @785nm, (b)) for a dimer of silver nanocubes (side length 75 nm), oriented edge to edge, inter-particle separation 2 nm, source polarized *parallel* to the inter-particle axis.

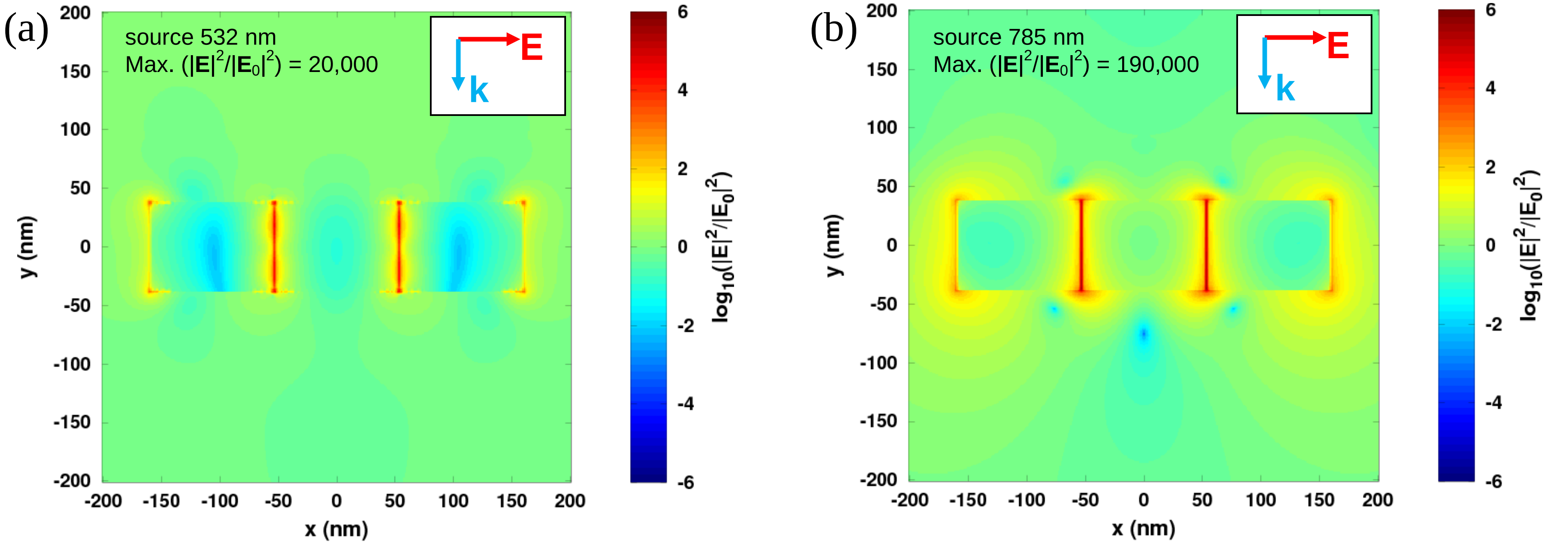

Supplementary Figure 4 - Trimer, edge-edge-edge orientation, 1nm separation. Spatial electric field enhancement distribution (@532nm, (a), and @785nm, (b)) for a trimer of silver nanocubes (side length 75 nm), in the edge-edge-edge orientation, inter-particle separation between each particle is 1nm, source polarized *parallel* to the inter-particle axis.

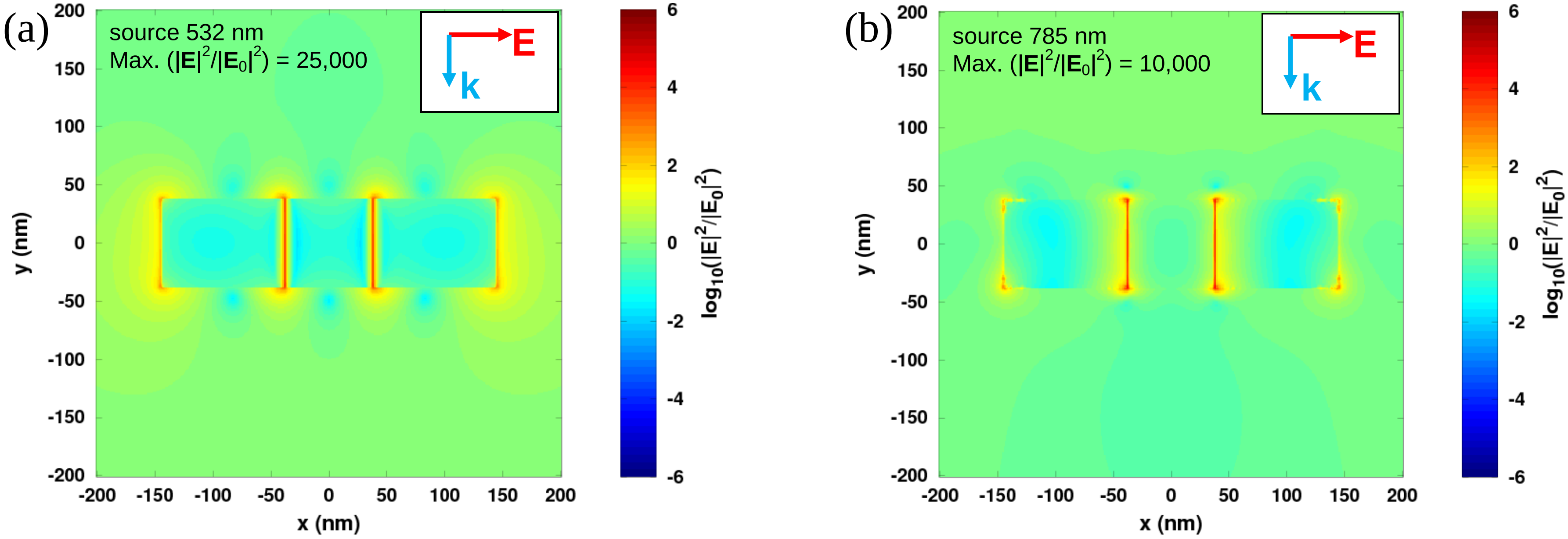

Supplementary Figure 5 - Trimer, edge-face-edge orientation, 1nm separation. Spatial electric field enhancement distribution (@532nm, (a), and @785nm, (b)) for a trimer of silver nanocubes (side length 75 nm), in the edge-face-edge orientation, inter-particle separation between each particle is 1nm, source polarized *parallel* to the inter-particle axis.

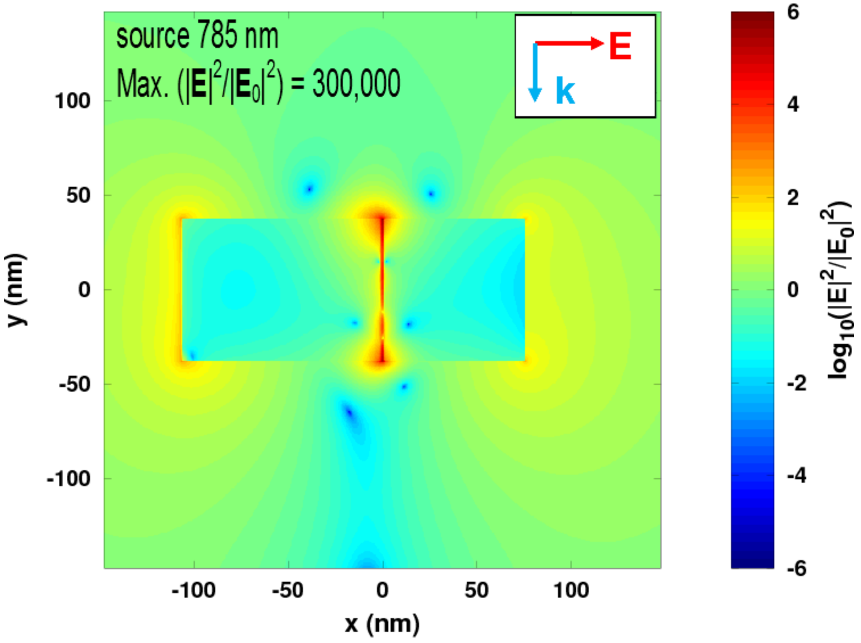

Supplementary Figure 6 – Investigation of field intensity calculation variations with decreasing mesh size. Spatial electric field intensity enhancement distribution @785nm for a dimer of silver nanocubes (side length 75 nm), in the face-to-edge orientation, inter-particle separation between the particles is 1nm, source polarized *parallel* to the inter-particle axis. In this simulation, the space between points of field intensity calculation was 0.5nm rather than 1nm in all other calculations.

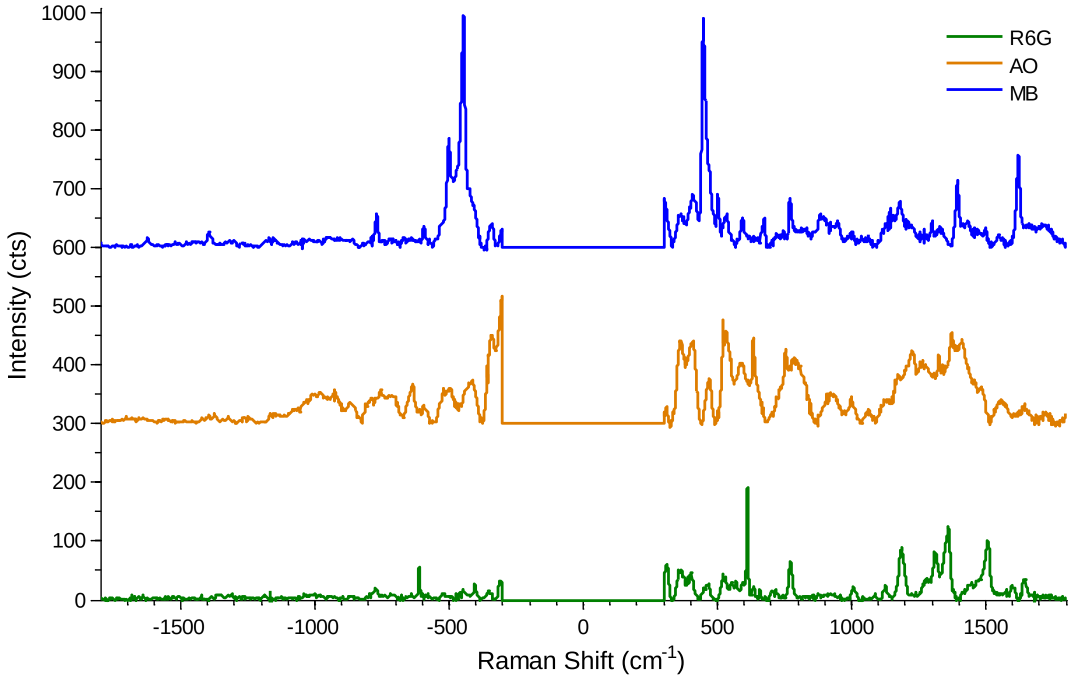

Supplementary Figure 7 - Combined anti-Stokes and Stokes spectra of MB, AO, and R6G on the Ag nanocube SERS platform. Upon visual inspection, we can observe that the anti-Stokes signals for the Ag-MB structure are much higher (in relation to their corresponding Stokes signals) than in the Ag-AO and Ag-R6G samples. This is true at both low energy shifts, where the MB anti-Stokes signal is actually greater than the Stokes, and at high energy shifts, where above 1000cm^{-1} only MB has measureable anti-Stokes signals.

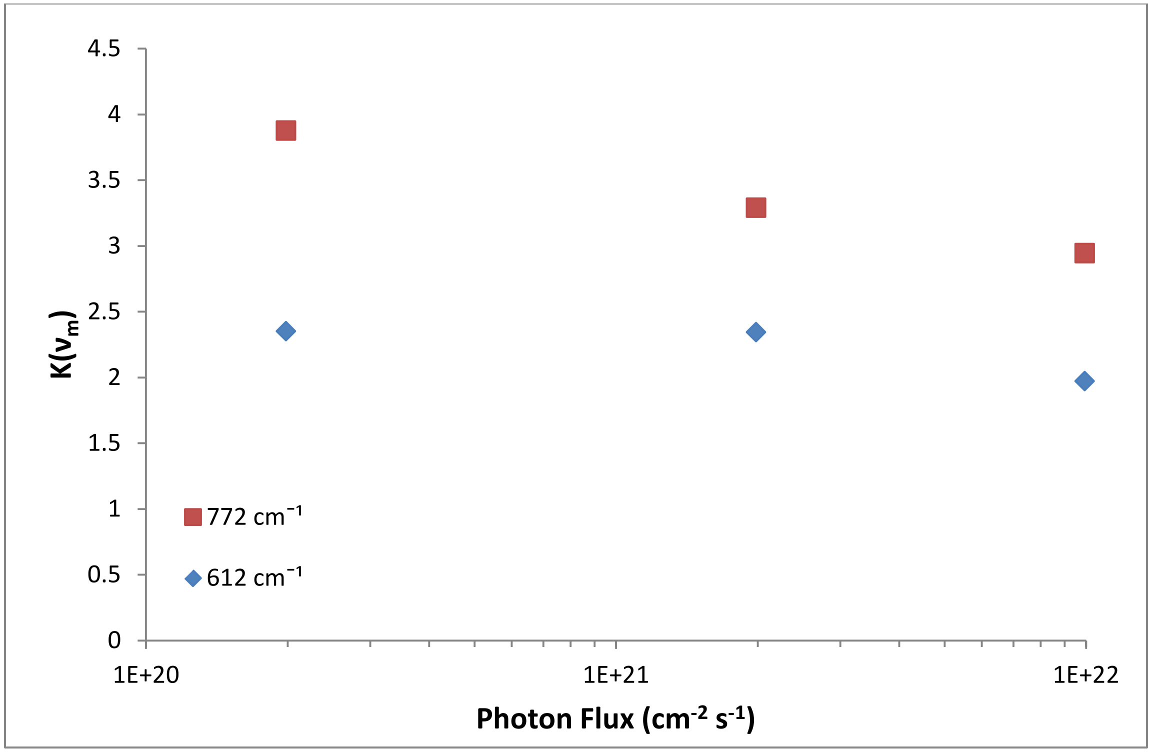

Supplementary Figure 8 – Intensity dependence of K values for vibrational modes of R6G. The strongest vibrational modes in terms of Raman signal under 785nm excitation show no increase with increasing intensity. The opposite behavior was observed for the MB-Ag system as discussed in the manuscript.

Supplementary Tables

	Face-Edge Dimer		Edge-Edge Dimer	
Source	separation 1 nm	separation 2 nm	separation 1 nm	separation 2 nm
532 nm	153.92	600.78	172.43	787.61
785 nm	23.56	87.99	27.2	199.63

Supplementary Table 1 – Average field intensities around dimers. Integrated values of ($|\mathbf{E}|^2/|\mathbf{E}_0|^2$) for dimers of silver nanocubes (side length 75 nm) in face-edge and edge-edge orientations, at inter-particle separations of 1 nm and 2 nm, under source wavelengths 532 nm and 785 nm. The values in this table are the average of the integrated field intensity at points located on the nanoparticle surfaces in each system. For each geometry and separation combination, the average enhancements under 532 nm and 785 nm were within an order of magnitude of each other.

	Edge-Edge-Edge Trimer	Edge-Face-Edge Trimer
Source	separation 1 nm	separation 1 nm
532 nm	220.68	470.26
785 nm	740.69	313.65

Supplementary Table 2 – Average field intensities around trimers. Integrated values of ($|\mathbf{E}|^2/|\mathbf{E}_0|^2$) for trimers of silver nanocubes (side length 75 nm) in edge-edge-edge and edge-face-edge orientations, at inter-particle separations of 1 nm, under source wavelengths 532 nm and 785 nm. The values in this table are the average of the integrated field intensity at points located on the nanoparticle surfaces in each system. For each geometry and separation combination, the average enhancements under 532 nm and 785 nm were within an order of magnitude of each other.

Ag-MB	
peak (cm^{-1})	K
447	3.91
771	7.67
1394	27.75
1621	34.45

Ag-AO	
peak (cm^{-1})	K
521	1.13
634	2.27
754	3.36

Ag-R6G	
peak (cm^{-1})	K
403	1.37
612	1.33
772	2.43
1008	8.08

Supplementary Table 3 – Numerical comparison of calculated K values for MB, AO, and R6G. Here we see quantitative evidence that the anti-Stokes signals, and thus K values, for the Ag-MB system are much more elevated than for the other probe molecules R6G and AO. Elevated K values in the other two molecules can be attributed to uneven LSPR enhancement of the anti-Stokes and Stokes scattered photons, which will be independent of the probe molecule, explaining why the K values for similar energy Raman shifts in the two molecules are similar. The additional anti-Stokes enhancement in Ag-MB is in this case a key signature of charge excitation.

Supplementary Note 1 – Additional FDTD simulations and field integrations

In supplementary figures 1-6 and supplementary tables 1 & 2, we present a number of additional FDTD simulations of Ag cube aggregates to supplement the face-edge dimers presented in the main text and further the understanding of the field intensity behaviors in ideal systems similar to our experimental system used in SERS measurements. The number of Ag particles, their relative orientations, and their separation distances were all individually varied in the numerous simulations. We find the field intensities behave as expected based on the prior more thorough works of Hao and Schatz[1]. Maximum field intensities vary from the order of $10^3 – 10^5$. The average field intensities around the surface of the particles are found to vary little (1 order of magnitude or less) throughout the simulations. We also find the overall behavior of the simulated field intensities does not change when the space between points of calculation is 0.5nm rather than the 1nm used in all calculations unless otherwise noted.

Supplementary Note 2 - Comparison between MB and other probe molecules under 785nm excitation

To rule out alternative explanations for the observance of the signatures of charge excitation in the Ag-MB system, we ran identical SERS measurements using the same Ag nanocube SERS platform and two other probe molecules, rhodamine 6G (R6G) and acridine orange (AO) (see Supplementary Figure 7). Both are common probe molecules used in Raman spectroscopy and neither exhibit any resonant behavior at 785nm that would result in resonance Raman effects. If the unusually high anti-Stokes signals from the Ag-MB measurements were a result of uneven LSPR enhancement of the anti-Stokes and Stokes scattered photons after emission from the molecule and not charge excitation, we would expect to observe the same signatures with any adsorbate molecule. Instead we find the K values of R6G and MB at 785nm excitation to be much lower than that of MB (see Supplementary Table 2). We additionally find no linear dependence of K on incident source intensity for R6G, a result that mirrors one presented by Haslett et al.[2] (see Supplementary Figure 8).

Supplementary References

1. Hao, E. & Schatz, G. C. Electromagnetic fields around silver nanoparticles and dimers. *J. Chem. Phys.* **120,** 357–66 (2004).

2. Haslett, T. L., Tay, L. & Moskovits, M. Can surface-enhanced Raman scattering serve as a channel for strong optical pumping? *J. Chem. Phys.* **113,** 1641 (2000).
